# Risk Factors of Histopathological Crescent Formation in Pediatric IgA Vasculitis Nephritis

**DOI:** 10.3390/medicina61081421

**Published:** 2025-08-06

**Authors:** Yanyan Jin, Yi Xie, Qian Lin, Yu Zhu, Limin Huang, Yang He, Haidong Fu

**Affiliations:** 1 Department of Nephrology, The Children’s Hospital, Zhejiang University School of Medicine, National Clinical Research Center for Child Health, Hangzhou 310052, China; 2 Department of Traditional Chinese Medicine, The Children’s Hospital, Zhejiang University School of Medicine, National Clinical Research Center for Child Health, Hangzhou 310052, China; 3 Department of Pediatric, Jinhua Maternal & Child Health Care Hospital, Jinhua 321000, China

**Keywords:** IgA vasculitis nephritis, histopathological crescent, childhood, glomerular sclerosis

## Abstract

*Background and Objectives*: This study aimed to explore the risk factors of histopathological crescent formation in pediatric IgA vasculitis nephritis (IgAVN). *Materials and Methods*: Enrolled patients with biopsy-proven IgAVN from Zhejiang University’s hospital were split into two groups: 377 with no crescents on histopathology (Group 1) and 364 with crescentic nephritis (Group 2). Collected data included clinical features, lab indicators, histopathological grading, and factors causing glomerular sclerosis. Logistic regression was used to assess factors affecting crescent formation in IgAVN. Double-immunofluorescence assay was used to detect TGF-β1, MCP-1, α-SMA, Collagen I, and FN1 in kidney biopsy specimens. The relationship between kidney fibrosis factors and histopathological grade were analyzed using Chi-square and Pearson tests. *Results*: A total of 741 patients with IgAVN were included in the study. Univariate logistic regression identified potential factors related to crescent formation, including age, gender, clinical classification, hematuria grade, 24 h urine protein level, peripheral white blood cells (WBCs), serum albumin, Cystatin-C, APTT, and PT. Multivariate analysis revealed statistical significance for age, 24 h urine protein, and WBCs across pathological grades (*p* < 0.05). Mantel–Haenszel Chi-square tests indicated a linear relationship between IgAVN pathological grade and α-SMA, TGF-β1, MCP-1, and FN1. Pearson correlation analysis confirmed a positive correlation between pathological grade and these markers. *Conclusions*: Age, 24 h urinary protein, and blood WBCs are identified as risk factors for histopathological crescent formation in children with IgAVN. Additionally, a higher pathological grade is associated with more pronounced fibrosis indicators.

## 1. Introduction

IgA vasculitis (IgAV) is the foremost type of vasculitis affecting children, characterized by its impact on various organs, with the skin, gastrointestinal tract, joints, and kidneys being the most frequently involved sites [1,2]. The reported annual incidence rate of IgA vasculitis (IgAV) varies between 6.21 cases per 100,000 children and 55.5 cases per 100,000 children across different nations [3,4]. Kidney involvement, referred to as IgA vasculitis nephritis (IgAVN), manifests in approximately 18.5% to 54% of patients diagnosed with IgAV [5,6,7]. It is one of the most frequently encountered secondary glomerular diseases in pediatric patients. Previously, it was believed that pediatric IgAVN is self-limiting and associated with good prognosis, but more recently it was found that some proportion of patients (1–7%) may progress to ESRD [8,9]. For children with moderate to severe proteinuria, 10% to 20% can progress to chronic kidney disease [10,11].

Previous research has indicated that an increased formation of crescents, along with higher pathological grades (ISKDC grades III-V), severe tubulointerstitial changes in the kidney, and the deposition of electron-dense material beneath the epithelium, are correlated with a poor prognosis [12,13,14,15,16]. A meta-analysis conducted in 2019 indicated that children suffering from IgAVN exhibited a later onset age, a decreased glomerular filtration rate, and initial renal manifestations indicative of nephrotic syndrome. Additionally, the kidney biopsy results (ISKDC grade III-V) revealed crescentic nephritis, which could serve as a predictor of an unfavorable prognosis [17]. Among them, a kidney biopsy revealing crescentic nephritis (ISKDC grade III-V) serves as a significant and independent predictor of unfavorable prognosis. Furthermore, an increased percentage of crescent formation correlates with a heightened tendency towards poor prognosis in patients with IgAVN. However, the factors that influence crescent formation in pediatric patients with IgAVN have not been conclusively determined. A study involving 191 pediatric patients with IgAVN revealed that the urinary white blood cell (WBC) count and the urinary microalbumin/creatinine ratio were identified as independent risk factors for the development of crescents [18].

Kidney fibrosis, particularly tubulointerstitial fibrosis, is characterized by excessive deposition of extracellular matrix (ECM) in the renal tubulointerstitial tissue. This represents the ultimate common pathway leading to almost all progressive cases of chronic kidney disease (CKD) [19]. Kidney fibrosis is a reliable prognostic indicator and a major determinant of kidney dysfunction [20]. Transforming growth factor β1 (TGF-β1), monocyte chemoattractant protein-1 (MCP-1) expression, alpha-smooth muscle actin (α-SMA), collagen type I (COL1/collagen I), and fibronectin-1 (FN1) constitute pivotal factors in renal fibrosis. Our previous research has demonstrated that the expression of TGF-β1 and monocyte chemoattractant protein-1 (MCP-1) is correlated with the progression of tubulointerstitial fibrosis in IgA vasculitis nephritis (IgAVN) [21]. At present, no reports have been published regarding the correlation between kidney fibrosis factors and histopathological grade in pediatric patients with IgAVN.

Consequently, it is essential to comprehend the risk factors associated with the development of crescents and their connection to fibrosis factors, for the purpose of enhancing the accuracy of prognostic predictions.

## 2. Materials and Methods

### 2.1. Patients

A retrospective analysis was conducted on 741 children diagnosed with IgA vasculitis nephropathy (IgAVN) who were admitted to the Department of Nephrology at the Children’s Hospital of Zhejiang University, spanning from November 2014 to March 2024. The validated EULAR/PRINTO/PRES criteria for the diagnosis of IgAV were used [22]. Kidney pathological findings were classified according to the International Society of Children’s Nephrology (ISKDC) classification. The design of this retrospective study and the methods used were approved by the ethics committee of the Children’s Hospital of Zhejiang University School of Medicine (2021-IRB-118).

### 2.2. Procedures

Demographic details, clinical information, and laboratory data were documented in the hospital records. Nephritis (kidney involvement) is defined as any kidney-related occurrence that arises during the course of the disease. This encompasses the presence of gross or microscopic hematuria, whether accompanied by proteinuria or not. Hematuria is characterized by a count exceeding five red blood cells per high-power microscopic field in a centrifuged urine specimen. Proteinuria is defined as the detection of a minimal amount of protein (+) via dipstick testing, or as proteinuria exceeding 150 mg as determined by a 24 h urine collection.

Kidney biopsy was recommended in cases of persistent proteinuria, persistent hematuria, nephropathy, nephrotic syndrome, or the coexistence of hematuria with kidney failure. All patients exhibited at least one of these abnormalities and underwent kidney biopsy, with the biopsy conducted between 12 days and 5 years following the initial manifestation of kidney-related symptoms. In all instances, IgAVN accompanied by diffuse IgA deposition in the mesangium was confirmed. The glomerular alterations were categorized based on a classification system devised by the ISKDC [23], as follows: (1) Grade I: minimal alterations; (2) Grade II: mesangial proliferation; (3) Grade III: focal or diffuse proliferation or sclerosis with <50% crescents; (4) Grade IV: focal or diffuse mesangial proliferation or sclerosis with 50–75% crescents; (5) Grade V: focal or diffuse mesangial proliferation or sclerosis with >75% crescents; (6) Grade VI: membranoproliferative-like lesions. Tubulointerstitial lesions, tubular atrophy, and interstitial fibrosis were semi-quantitatively assessed and categorized into the following four grades: none (denoted as -), mild (denoted as +), moderate (denoted as ++), or severe (denoted as +++).

Patients were divided into two groups based on pathological grade: Group 1: IgA vasculitis nephritis without crescents (ISKDC grade ≤ II) or Group 2: IgA vasculitis nephritis with crescents (ISKDC grade ≥ III). Hematuria grade: mild hematuria (5/HP ≤ RBC < 40/HP), moderate hematuria (40/HP ≤ RBC < 100/HP), or severe hematuria (RBC ≥ 100/HP). Proteinuria grade: mild proteinuria (<25 mg/kg/24 h); moderate proteinuria (≥25 mg/kg/24 h and <50 mg/kg/24 h); or severe proteinuria (≥50 mg/kg/24 h). Clinical classification: isolated proteinuria, isolated hematuria, rapidly progressive glomerulonephritis acute nephritis, chronic nephritis, nephrotic syndrome, and hematuria with proteinuria.

To enhance the interpretability of the study results, the present research stratified certain continuous variables into ordered hierarchical variables. The grading system and the corresponding assignments for these variables are presented in Table 1, which outlines the original data and their respective SPSS (Version 27.0) variable assignments.

### 2.3. Semiquantitative Immunofluorescence Analysis

Twenty-three paraffin samples were employed in the study, six of which belonged to Group 1, twelve to Group 2, and five normal renal specimens exhibiting hematuria accompanied by mild pathological changes which were selected to constitute the control group. A paraffin section double-immunofluorescence assay was utilized to detect and observe the kidney fibrosis factors TGF-β1, MCP-1, α-SMA, Collagen I, and FN1 in the various specimens (groups). Under UV excitation the nucleus stained with DAPI appeared blue, and the presence of corresponding fluorescence labeled with either red or green light indicated a positive result. Using Image Pro plus 6.0 (Media Cybernetics, Inc., Rockville, MD, USA) software, we selected the same red and green colors and use a unified positive standard to analyze the positive area, tissue area, and positive rate of each photo. 20.0X is CY3; 20.0X-2 is FITC. According to the staining range, there are four levels: negative (-): no positive staining; weak positive (+): green positive range 0–25%; positive (++): green positive range of 26–50%; strong positive (+++): the green positive range is over 50%.

### 2.4. Statistical Analysis

All statistical analyses were performed utilizing the R3.6.3 software package. Continuous variables were represented by their median and interquartile range (IQR), with *t*-tests or non-parametric tests employed to compare differences between two groups. Categorical variables were expressed in terms of frequency and percentage and analyzed using either Pearson’s Chi-square test or Fisher’s exact test. Logistic regression analysis was utilized to explore the risk factors associated with IgAVN pathological grading. With IgAVN pathological grading serving as the dependent variable, univariate logistic regression analysis was initially conducted to screen for potential risk factors. Subsequently, significant variables (*p* < 0.1) from the univariate logistic regression analysis were incorporated into multivariate logistic regression analysis to identify risk factors related to IgAVN pathological grading. The Mantel–Haenszel Chi-square test was employed to determine the presence of a linear relationship between the pathological grade of IgAVN in children and kidney fibrosis factors. The pathological grade of IgAVN patients ranged from 0 to 1, while the kidney fibrosis factor grade ranged from 1 to 3. Pearson’s analysis was used to assess correlation.

This study used GraphPad Prism 7.0 software (GraphPad Software, CA, USA) for plotting.

## 3. Results

### 3.1. Demographic Characteristics in Children with IgAV Nephritis

This retrospective study encompassed 741 patients who were diagnosed with IgAVN based on comprehensive clinical and pathological assessments, as detailed in Table 2. Specifically, Group 1 comprised a total of 377 patients, with a mean age of 8.6 ± 2.9 years. Within this group, there were 212 males (representing 56.2% of the cohort) and 165 females (constituting 43.8% of the cohort). Group 2 consisted of 364 patients, with a mean age of 9.4 ± 3.0 years. This group included 216 males (59.3% of the cohort) and 148 females (40.7% of the cohort). The comparison between Group 1 and Group 2, as outlined in Table 2, is as follows: (1) clinical classification: 38 cases of simple proteinuria (10.1%) vs. 11 cases (3.0%), 23 cases of simple hematuria (6.1%) vs. 6 cases (1.6%), 1 case of progressive nephritis (0.3%) vs. 1 case (0.3%), 4 cases of acute nephritis (1.1%) vs. 0 cases (0.0%), 0 cases of chronic nephritis (0.0%) vs. 1 case (0.3%), 22 cases of nephrotic syndrome (5.8%) vs. 54 cases (14.8%), and 289 cases of hematuria and proteinuria (76.7%) vs. 291 cases (79.9%); (2) digestive tract involvement: 171 cases with none (45.7%) vs. 168 cases (46.4%), 146 cases (39.0%) with but without gastrointestinal bleeding vs. 132 cases (36.5%), and 57 cases (15.2%) with combined gastrointestinal bleeding vs. 62 cases (17.1%); (3) kidney involvement time (months): <1 month, 303 (80.4%) vs. 296 (81.3%), from January to June, 63 (16.7%) vs. 55 (15.1%), and >June 11 (2.9%) vs. 13 (3.6%); (4) hematuria grading: 52 cases with none (13.8%) vs. 15 cases (4.1%), mild 175 cases (46.4%) vs. 169 cases (46.4%), moderate 70 cases (18.6%) vs. 75 cases (20.6%), and severe 80 cases (21.2%) vs. 105 cases (28.8%); (5) urinary protein grading: mild 165 cases (43.8%) vs. 108 cases (29.7%), and 118 cases (31.3%) were severe compared to 156 cases (42.9%); (6) laboratory examination: Blood WBC (×10^9^/L) 9.2 ± 4.3 vs. 10.4 ± 4.4, PLT (×10^9^/L) 323.0 ± 87.3 vs. 317.2 ± 92.4, ALB (g/L) 38.9 ± 5.5 vs. 37.0 ± 6.9, Scr (μmol/L) 52.7 ± 51.9 vs. 52.4 ± 18.6, BUN/Scr 4.6 ± 2.5 vs. 4.9 ± 4.2, UA (μmol/L) 266.9 ± 89.4 vs. 281.9 ± 189.6, Cys-C (mg/L) 0.8 ± 0.3 vs. 0.9 ± 0.3, APTT (seconds) 27.0 ± 4.5 vs. 26.4 ± 4.6, and PT (seconds) 11.0 ± 1.1 vs. 10.7 ± 0.9.

### 3.2. Correlation Between Clinical, Laboratory Parameters and Crescents

The results of the univariate logistic regression analysis indicated that age, gender, clinical classification, hematuria severity, urine protein severity, blood WBC levels, ALB levels, Cys-C levels, APTT, and PT may be associated with the development of crescents. Conversely, factors such as gastrointestinal involvement, duration of kidney involvement, PLT levels, BUN/Scr ratios, and UA levels were found to be unrelated to the pathological grade, as presented in Table 3.

The results of the multivariate logistic regression analysis, with other confounding factors controlled, indicated that age (*p* = 0.001, OR = 1.133, 95% CI: 1.072–1.197), urinary protein grade, and blood WBC count (*p* = 0.002, OR = 1.067, 95% CI: 1.024–1.113) were significant factors influencing crescent formation in IgAVN. Please refer to Table 4 for details.

### 3.3. Evaluation and Verification of the Influencing Factors

**Figure 1:**
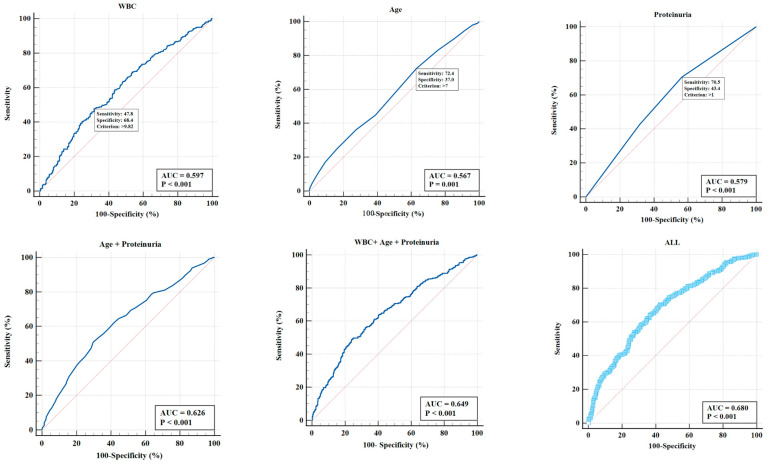
The ROC curve of the *p*-m value was constructed using Medcalc Version 20, and the discrimination of the IgAVN influencing factors were evaluated using the AUC. The ROC curve showed that the AUC of the *p*-m value in age, urinary protein grade, and blood WBC count was 0.649. Upon incorporating all indicators that exhibited statistical significance in the univariate regression analysis, the AUC attained a value of 0.68, including age, gender, clinical classification, hematuria severity, urine protein severity, blood WBC levels, ALB levels, Cys-C levels, APTT, and PT.

Utilizing Medcalc Version 20, the ROC curve pertaining to the *p*-m value was generated, and the area under this curve (AUC) served as a metric to evaluate the predictive capability of various IgAVN influencing factors. As illustrated in Figure 1, the ROC curve demonstrated an AUC of 0.649 for the *p*-m value in relation to age, urinary protein grade, and blood WBC count. Upon incorporating all indicators that exhibited statistical significance in the univariate regression analysis, the AUC attained a value of 0.68, including age, gender, clinical classification, hematuria severity, urine protein severity, blood WBC levels, ALB levels, Cys-C levels, APTT, and PT.

### 3.4. Immunofluorescence Comparison of Pathological Tissue Slices for Kidney Fibrosis Indicators

**Figure 2:**
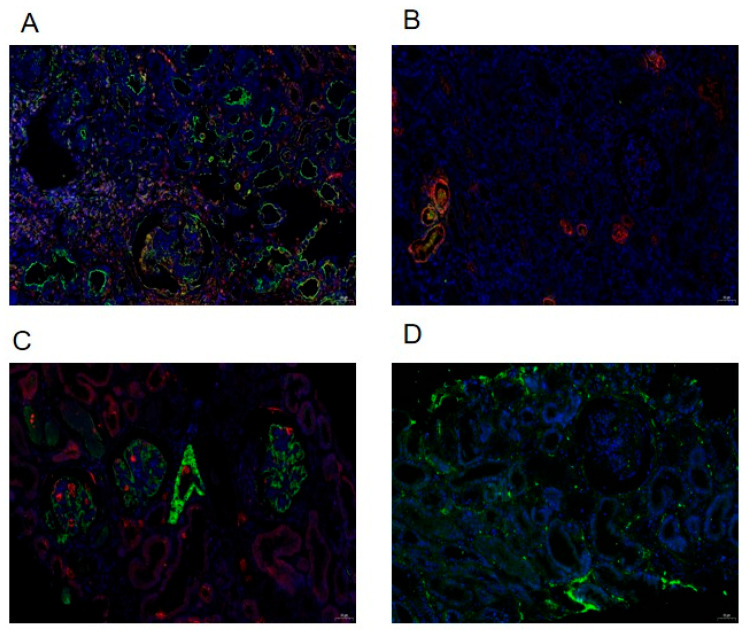
Using paraffin section double-immunofluorescence assay to detect and observe kidney fibrosis factors in kidney specimens. TGF-β1 (**A**), MCP-1 (**B**), α-SMA (**C**), and FN1 (**D**) with positive expression indicated by corresponding fluorescent-labeled with red light (CY3 labeling) or green light (FITC labeling).

To assess the extent of kidney fibrosis among patients exhibiting various pathological grades, we employed immunofluorescence staining analysis and conducted semi-quantitative scoring on kidney biopsy samples. This analysis was conducted to evaluate the kidney fibrosis factors TGF-β1, MCP-1, α-SMA, Collagen I, and FN1 in distinct sample groups (groups). Positive expression was indicated by the presence of corresponding fluorescent labeling, specifically red light (CY3 labeling) or green light (FITC labeling), as exemplified in the representative images presented in Figure 2. Preliminary experimental results indicated an absence of Collagen I expression, leading to the termination of subsequent experimental procedures. Conversely, α-SMA, TGF-β1, MCP-1, and FN1 were all expressed in the kidney tissue of patients with IgAVN.

The mild damage group exhibited weak positivity (+) and positivity (++); whereas the moderate to severe damage group predominantly showed positivity (++) and strong positivity (+++). The Mantel–Haenszel Chi-square test results indicated a correlation between the pathological grade of IgAVN patients and α-SMA (χ^2^ = 5.844, *p* < 0.05), TGF-β1 (χ^2^ = 6.800, *p* < 0.05), MCP-1 (χ^2^ = 5.183, *p* < 0.05), and FN1 (χ^2^ = 6.476, *p* < 0.05), all of which exhibited a linear relationship with the ISKDC grade. The Pearson correlation analysis further confirmed a significant correlation between these factors and the ISKDC grade, specifically α-SMA (R = 0.586, *p* < 0.05), TGF-β1 (R = 0.632, *p* < 0.05), MCP-1 (R = 0.552, *p* < 0.05), and FN1 (R = 0.617, *p* < 0.05). All these factors increased in conjunction with the elevation of the pathological grade, as illustrated in Table 5.

## 4. Discussion

IgAVN continues to be a prevalent secondary glomerular disease in childhood, posing a significant threat to children’s health. Since its inception in 1977, the ISKDC’s pathological grading diagnostic standard for IgAVN has been widely adopted and remains the most commonly utilized standard, serving as the primary reference for selecting diagnosis and treatment plans [24]. Although there are different studies on the relationship between crescent and prognosis, overall research suggests that crescent remains a pathological indicator of the severity of poor prognosis in IgAVN [17,25].

A large-scale multicenter retrospective study abroad found that 97% of HSP patients experienced kidney damage within 6 months of onset, with 85% occurring within 4 weeks after onset [8]; in 2013, a multicenter survey in China found that 73.4% of children had kidney damage within 4 weeks of onset, and 96.7% had kidney damage within 6 months of onset [26]. The results of 741 children with IgAVN in our center showed that 80.4% of the mild damage group occurred within 4 weeks; 97.1% occurred within 6 months; 81.3% of the moderate to severe injury group occurred within 4 weeks; and 96.4% occurred within 6 months, which is consistent with previous research. The age of mild damage is 8.6 ± 2.9 years old, and the age of moderate to severe damage group is 9.4 ± 3.0 years old. Univariate and multivariate logistic regression analysis showed that age is related to pathological grade, and the older the age, the more severe the pathological grade, consistent with previous studies [17,25]. The presence or absence of gastrointestinal bleeding is not related to the pathological grade. The clinical classification of the mild impairment group is mainly hematuria and proteinuria, followed by simple proteinuria and nephrotic syndrome. The group with moderate to severe damage mainly consists of hematuria and proteinuria, followed by nephrotic syndrome type and simple proteinuria. Univariate logistic regression analysis showed that clinical classification of nephrotic syndrome had a heavier pathological grading, but multivariate logistic regression analysis showed that clinical classification was not correlated with pathological grading. This study graded hematuria according to the number of red blood cells under high magnification of urine routine. Univariate logistic regression analysis showed that patients with severe hematuria had a heavier pathological grade, but multivariate logistic regression analysis showed that the degree of hematuria was not correlated with pathological grade. Assadi F [27] found that the clinical presentation of nephrotic syndrome at onset is an independent predictor of the severity of glomerular disease. This study demonstrated that children exhibiting a high level of urine protein were more inclined to be classified as having a pathological grade above III, and such protein levels were identified as independent risk factors, which is in accordance with the existing literature [27,28,29]. Feng D et al. found that serum albumin and IgG levels were significantly reduced in IgAVN patients with nephrotic proteinuria, while blood urea nitrogen and cystatin C levels increased [28]. Lu S et al. found that 24 h total urine protein, serum uric acid, cystatin C (Cys-C), and β2-microglobulin levels were positively correlated with pathological grading and activity [29]. The clinical characteristics are related to the severity of kidney pathology. In this study, univariate logistic regression analysis found that there were statistically significant differences in blood WBC, ALB, Cys-C, APTT, and PT in laboratory tests (all *p* < 0.05); however, multivariate logistic regression analysis showed that only blood WBC was correlated with pathological grading. Overall, age, 24 h urine protein quantification, and blood WBC are independent risk factors for moderate to severe pathological damage. A recent similar study [18] included 191 patients to study the influencing factors of crescent formation, and the results showed that higher urinary white blood cell (WBC) count and higher urinary microalbumin/creatinine ratio were independent risk factors for crescent formation in IgAVN. Perhaps due to different inclusion criteria and varying number of cases, it is inconsistent with the conclusions of this study. In this study, the AUC for the IgAVN influencing factors, which include age, urinary protein grade, and blood white blood cell (WBC) count, was determined to be 0.649. Upon incorporating all indicators that demonstrated statistical significance in the univariate regression analysis, the AUC increased to 0.68, indicating that the model possesses a moderate level of discriminatory capability. This model holds significant value for individuals who are unable to undergo kidney biopsies or require an initial assessment of disease severity at the onset of illness. Nevertheless, our research reveals disparities among clinical manifestations, laboratory examinations, and pathological outcomes, particularly regarding the lack of a direct correlation between the severity of urinary protein and the pathological grade. Consequently, it is advised that a kidney biopsy be conducted when circumstances allow.

Kidney fibrosis serves as a dependable prognostic marker, and it is essential to determine if heightened pathological grades are linked to more severe fibrosis. As such, a thorough investigation into the expression of TGF-β1, MCP-1, α-SMA, and FN1 within the renal tissues of individuals suffering from IgAVN was undertaken. Concurrently, a linear and positive correlation exists between the pathological grade of IgAVN patients and these fibrosis factors. Kawasaki Y et al. found that IgAVN patients with crescent formation had glomeruli and stroma during the first biopsy and that α-SMA staining was significantly higher than that of IgAVN patients without crescent formation, and in the second biopsy the glomerulus and stroma were significantly higher and the α-SMA was relatively high. Furthermore, in IgAVN patients with poor prognosis these are higher than in IgAVN patients with good prognosis, indicating that they may be related to the progression of IgAVN kidney injury, consistent with our research. As such, α-SMA is an indicator of the severity of IgAVN disease and may be one of the prognostic indicators [30]. Wang J et al. found that the increase in 24 h urine MCP-1 levels in IgAVN patients is parallel to the increase in total urine protein [31]. Fuentes Y et al. found that early urinary MCP-1/creatinine levels were elevated in severe IgAVN patients [32]. Shuiai Z et al. found that the degree of kidney tubulointerstitial fibrosis is related to TGF β1 and MCP-1 [21]. Based on our findings, the study indicates that the occurrence of kidney fibrosis is more probable following the formation of crescents.

The objective of undertaking a small-sample study on fibrosis indicators is to preliminarily ascertain if elevated pathological grades are associated with a greater severity of fibrosis. It is imperative to acknowledge that the sample size pertaining to the fibrosis factors in the sub-study was constrained, and subsequent research initiatives will endeavor to augment the sample size to more conclusively elucidate the interrelationships between fibrosis indicators, crescent formation, and pathological grades. The AUC of our model is 0.68, indicating a moderate ability to distinguish between positive and negative samples, albeit with a relatively modest effect size. Nonetheless, further research is necessary to conduct a more comprehensive analysis of the factors associated with crescent formation.

## 5. Conclusions

In summary, according to the findings derived from both univariate and multivariate logistic regression analyses, age, 24 h urine protein levels, and blood WBC counts have been established as independent factors influencing the formation of histological crescents in pediatric patients with IgAVN. Additionally, a heightened pathological grade is associated with more significant fibrosis indicators.

## Figures and Tables

**Table 1:** Original data and corresponding SPSS variable assignments.

Variable	Value	Set
Kidney involvement time	≤4 weeks	0
	4 weeks~6 months	1
	>6 months	2
Urinary routine red blood cells (under high magnification)	0	0
	RBC > 5 but <40	0
	RBC 40–100	1
	RBC > 100	2
24 h urine protein quantification(mg/kg/d)	<25	0
	25–50	1
	>50	2
ISKDC grade	≤II	0
	≥III	1
Kidney tubulointerstitial injury	+	0
	++∼++++	1
Digestive tract involvement	None	0
	With but without gastrointestinal bleeding	1
	Combined gastrointestinal bleeding	2

**Table 2:** Description of study population with and without crescent formation.

Variable	Group 1	Group 2	*p*-Value
n	377	364	
Age	8.6 ± 2.9	9.4 ± 3.0	0.002
Gender			0.392
Male	212 (56.2%)	216 (59.3%)	
Female	165 (43.8%)	148 (40.7%)	
Clinical classification			<0.001
isolated proteinuria	38 (10.1%)	11 (3.0%)	
isolated hematuria	23 (6.1%)	6 (1.6%)	
rapidly progressive glomerulonephritis	1 (0.3%)	1 (0.3%)	
acute nephritis	4 (1.1%)	0 (0.0%)	
chronic nephritis	0 (0.0%)	1 (0.3%)	
nephrotic syndrome	22 (5.8%)	54 (14.8%)	
hematuria with proteinuria	289 (76.7%)	291 (79.9%)	
Digestive tract involvement			0.689
none	171 (45.7%)	168 (46.4%)	
with but without gastrointestinal bleeding	146 (39.0%)	132 (36.5%)	
with combined gastrointestinal bleeding	57 (15.2%)	62 (17.1%)	
Kidney involvement time (month)			0.755
<1	303 (80.4%)	296 (81.3%)	
1~6	63 (16.7%)	55 (15.1%)	
>6	11 (2.9%)	13 (3.6%)	
Hematuria grading			<0.001
none	52 (13.8%)	15 (4.1%)	
mild	175 (46.4%)	169 (46.4%)	
moderate	70 (18.6%)	75 (20.6%)	
severe	80 (21.2%)	105 (28.8%)	
Urinary protein grading			<0.001
mild	165 (43.8%)	108 (29.7%)	
moderate	94 (24.9%)	100 (27.5%)	
severe	118 (31.3%)	156 (42.9%)	
WBC (×10^9^/L)	9.2 ± 4.3	10.4 ± 4.4	<0.001
PLT (×10^9^/L)	323.0 ± 87.3	317.2 ± 92.4	0.352
ALB (g/L)	38.9 ± 5.5	37.0 ± 6.9	<0.001
Scr (μmol/L)	52.7 ± 51.9	52.4 ± 18.6	0.024
BUN/Scr	4.6 ± 2.5	4.9 ± 4.2	0.033
UA (μmol/L)	266.9 ± 89.4	281.9 ± 189.6	0.166
Cys-C (mg/L)	0.8 ± 0.3	0.9 ± 0.3	0.049
APTT (s)	27.0 ± 4.5	26.4 ± 4.6	0.05
PT (s)	11.0 ± 1.1	10.7 ± 0.9	0.002

WBC, white blood cell; PLT, platelet; ALB, albumin; Scr, serum creatinine; BUN, blood urine nitrogen; UA, uric acid; Cys-C cystatin C; APTT, activated partial thromboplastin time; PT prothrombin time.

**Table 3:** Univariate logistic regression analysis of influencing factors of IgAVN pathological.

IgAVN Pathological Grading	b	SE	*p*	OR	95%CI of OR
Age (years)	0.089	0.025	<0.001	1.093	1.04–1.149
Gender					
Male				Ref	
Female	−0.127	0.149	0.392	0.880	0.658–1.179
Clinical classification					
isolated proteinuria				Ref	
isolated hematuria	−0.104	0.572	0.856	0.901	0.294–2.766
rapidly progressive glomerulonephritis	1.240	1.455	0.394	3.455	0.199–59.837
acute nephritis	−13.326	441.372	0.976	0.000	0-Inf
chronic nephritis	15.806	882.743	0.986	Inf	0-Inf
nephrotic syndrome	2.138	0.426	<0.001	8.479	3.682–19.53
hematuria with proteinuria	1.247	0.352	<0.001	3.479	1.744–6.939
Digestive tract involvement					
none				Ref	
with but without gastrointestinal bleeding	−0.083	0.162	0.608	0.920	0.67–1.264
with combined gastrointestinal bleeding	0.102	0.213	0.633	1.107	0.729–1.682
IgAVN pathological grading	b	SE	*p*	OR	95%CI of OR
Kidney involvement time (month)					
<1				Ref	
1–6	−0.112	0.202	0.578	0.894	0.602–1.327
>6	0.190	0.418	0.649	1.210	0.534–2.743
Hematuria grading					
none				Ref	
mild	1.208	0.312	0.000	3.348	1.815–6.174
moderate	1.312	0.337	0.000	3.714	1.919–7.189
severe	1.515	0.329	<0.001	4.550	2.39–8.663
Urinary protein grading					
mild				Ref	
moderate	0.486	0.190	0.010	1.625	1.121–2.357
severe	0.703	0.174	<0.001	2.020	1.437–2.84
WBC (×10^9^/L)	0.066	0.018	<0.001	1.068	1.03–1.107
PLT (×10^9^/L)	−0.001	0.001	0.378	0.999	0.998–1.001
ALB (g/L)	−0.050	0.012	<0.001	0.951	0.928–0.974
BUN/Scr (μmol/L)	0.006	0.007	0.361	1.006	0.993–1.019
UA (μmol/L)	0.001	0.001	0.432	1.001	0.999–1.002
Cys-C (mg/L)	0.504	0.263	0.055	1.655	0.989–2.77
APTT (s)	−0.029	0.016	0.072	0.971	0.941–1.003
PT (s)	−0.234	0.078	0.003	0.791	0.679–0.922

**Table 4:** Multivariate logistic regression analysis of influencing factors of different pathological grades.

IgAVN Pathological Grading	b	SE	*p*	OR	95%CI of OR
Age (years)	0.125	0.028	<0.001	1.133	1.072–1.197
Gender					
Male				Ref	
Female	−0.165	0.161	0.307	0.848	0.618–1.163
Clinical classification					
isolated proteinuria				Ref	
isolated hematuria	−0.282	0.682	0.679	0.754	0.198–2.872
rapidly progressive glomerulonephritis	−0.507	1.712	0.767	0.602	0.021–17.281
acute nephritis	−14.480	421.441	0.973	0.000	0-Inf
chronic nephritis	15.089	882.744	0.986	Inf	0-Inf
nephrotic syndrome	1.055	0.629	0.094	2.871	0.836–9.858
hematuria with proteinuria	0.665	0.510	0.192	1.945	0.716–5.282
Hematuria grading					
none				Ref	
mild	0.676	0.442	0.126	1.966	0.827–4.677
moderate	0.682	0.465	0.142	1.979	0.795–4.926
severe	0.873	0.460	0.058	2.393	0.971–5.898
Urinary protein grading					
mild				Ref	
moderate	0.371	0.213	0.081	1.449	0.955–2.197
severe	0.463	0.223	0.038	1.589	1.026–2.461
WBC (increase per 10^9^/L)	0.065	0.021	0.002	1.067	1.024–1.113
ALB (increase per 1 g/L)	−0.007	0.020	0.740	0.994	0.956–1.033
Cys-C (increase per 1 mg/L)	0.352	0.308	0.252	1.422	0.778–2.6
APTT (s)	0.002	0.020	0.929	1.002	0.963–1.043
PT (increase per 1 s)	−0.106	0.097	0.274	0.899	0.744–1.088

**Table 5:** Immunofluorescence staining of pathological tissue sections in IgAVN patients with and without crescent formation.

		Group 1 (n = 6)	Group 2 (n = 12)	*χ*^2^ (*p*)	*R* (*p*)
α-SMA	-	0	0	5.844 (0.016)	0.586 (0.011)
	+	3	1		
	++	3	5		
	+++	0	6		
**TGF-β1**	-	0	0	6.800 (0.009)	0.632 (0.005)
	+	4	1		
	++	2	6		
	+++	0	5		
**MCP-1**	-	0	0	5.183 (0.023)	0.552 (0.018)
	+	2	1		
	++	4	4		
	+++	0	7		
**FN1**	+	5	2	6.476 (0.011)	0.617 (0.006)
	++	1	6		
	+++	0	4		

Negative (-): no positive staining; weak positive (+): positive range 0–25%; positive (++): positive range of 26–50%; strong positive (+++): positive range is over 50%.

## Data Availability

The raw data supporting the conclusions of this article will be made available by the corresponding author Yanyan Jin, without undue reservation.

## Abbreviations

IgAV	IgA vasculitis
IgAVN	IgA vasculitis nephritis
TGF- β1	Transforming growth factor β1
MCP-1	Monocyte chemoattractant protein-1 expression
α-SMA	Alpha-smooth muscle actin
FN1	Fibronectin-1
IQR	Interquartile range
WBC	White blood cell
PLT	Platelet
ALB	Albumin
Scr	Serum creatinine
BUN	Blood urine nitrogen
UA	Uric acid
Cys-C	Cystatin C
APTT	Activated partial thromboplastin time
PT	Prothrombin time
